# Isosmotic Macrocation Variation Modulates Mineral Efficiency, Morpho-Physiological Traits, and Functional Properties in Hydroponically Grown Lettuce Varieties (*Lactuca sativa* L.)

**DOI:** 10.3389/fpls.2021.678799

**Published:** 2021-06-04

**Authors:** Giandomenico Corrado, Veronica De Micco, Luigi Lucini, Begoña Miras-Moreno, Biancamaria Senizza, Gokhan Zengin, Christophe El-Nakhel, Stefania De Pascale, Youssef Rouphael

**Affiliations:** ^1^Deparment of Agricultural Sciences, University of Naples Federico II, Portici, Italy; ^2^Department for Sustainable Food Process, Research Centre for Nutrigenomics and Proteomics, Università Cattolica del Sacro Cuore, Piacenza, Italy; ^3^Physiology and Biochemistry Research Laboratory, Department of Biology, Selcuk University, Konya, Turkey

**Keywords:** macronutrients, essential elements, nutrient utilization efficiency, leaf morphology, plant physiology, polyphenols, enzymatic activities

## Abstract

The management of mineral elements in agriculture is important for their nutritional role for plants and dietary value for humans, sparking interest in strategies that can increase mineral use efficiency and accumulation in plant food. In this work, we evaluated the effects of the isosmotic variations of the concentration on three macrocations (K, Ca, and Mg) in lettuce (*Lactuca sativa* L.). Our aim was to improve the nutritional components of this valuable dietary source of minerals. Using a full factorial design, we analyzed mineral utilization efficiency (UtE), leaf morphology, gas exchange parameters, phenolic profiles (through ultra-high performance liquid chromatography coupled to a quadrupole-time-of-flight (UHPLC-QTOF) mass spectrometry), and enzymatic activities in two phytochemically diverse butterhead lettuce varieties (red or green). Plants were fed in hydroponics with three nutrient solutions (NSs) with different ratios of K, Ca, and Mg. The variation of these minerals in the edible product was associated with alterations of the morphology and physiology of the leaves, and of the quality and functional properties of lettuce, with a trade-off between total accumulation and mineral UtE. Moreover, in non-limiting conditions of nutrient availability, significant mineral interactions were also present. The flexibility of the plant response to the different ratios of macrocations, and the observed large intraspecific variation, were adequate to provide mineral-specific phytochemical profiles to the edible product. Specifically, the full-red lettuce provided more interesting results in regard to the compositional and functional attributes of the leaves.

## Introduction

Plants, humans, and any other living organism need inorganic elements to carry out their life cycle, which leads to the definition of essential nutrients for those that are considered irreplaceable and necessary for the biochemical processes that support growth and reproduction (O'dell and Sunde, 1997). Autotrophic plants acquire minerals from the soils primarily as inorganic ions, thus representing the ultimate dietary source of minerals (Grusak, 2002). These are typically distinguished as macronutrients and micronutrients. The essential mineral elements for plants that are required in high quantities (i.e., N, P, K, Ca, Mg, and S) are classified as macronutrients. They have a relative concentration often between 0.2 and 4% of the total plant dry weight (DW) (Marschner, 2011). Humans, and more generally mammals, require more than 28 essential elements (Mcdowell, 2003). Physiological disorders in some populations or livestock are usually reported for the deficiency of Ca and Mg, within the macrominerals (i.e., those required between 100 and 1,000 mg/day), and Cu, Zn, I, and Se, within the trace elements (i.e., those required <10 mg/day) (Frossard et al., 2000; Mcdowell, 2003; White and Broadley, 2005; Broadley and White, 2010).

There is a growing interest in agronomic and breeding approaches to sustainably improve mineral efficiency in crops (Singh and Singh, 2019), with the aim of increasing yield while ensuring a higher accumulation of essential mineral per chemical input available in the environment (Grusak and Dellapenna, 1999; White and Brown, 2010). Mineral concentration in plants varies largely among species, growth stages, and organs. Staple crops are considered less efficient in terms of mineral content per dry matter (DM) (Poletti et al., 2004), and vegetables are the main high-density source of essential minerals for humans (White and Brown, 2010). It has been long proposed that the selection for yield, along with a widespread reliance on fertilizers, may have inadvertently altered the amount of mineral nutrients in the edible part of a plant (Davis, 2009; Marles, 2017). The inverse relationship between improvements in the DM content (represented mainly by carbohydrates such as starch and fiber) and those relative to the amount of mineral nutrients is called the “dilution effect.” Even if there is not a universal consensus on the prevalence and magnitude of this phenomenon (Davis, 2009; Marles, 2017), it has been observed that the most prominent apparent decline in minerals was observed in vegetables (Davis, 2009), with potential large-scale implications. Dietary surveys indicated that approximately 10% of the adult population that is energetically well-nourished (e.g., in developed countries such as the UK and USA) assumes a suboptimal intake of K, Mg, or Ca, and it is considered to be at risk of deficiency (Broadley and White, 2010). Even allowing for the potential pitfalls related to the analysis of historical data and of plant food of uncontrolled origin, there are two scientific counterarguments worth of investigation. The first is that there is a considerable variation within cultivated germplasm in the mineral concentrations of edible products (Guttieri et al., 2015). The intraspecific variability could be adequate to limit generalization on the overall decline in the quality of a single plant species (Guzmán et al., 2014). Therefore, efforts should be directed toward the selection and use of more nutrient-efficient plant varieties. The second is that the mineral quality of the plant is mainly dictated by nutrient availability and a lower nutrient efficiency may reflect an intensification of cropping systems, such as high nitrogen, phosphorus, and potassium (NPK) fertilization along with the impoverishment of trace elements in soil (Welch and Graham, 2002). Thus, it is necessary to balance and adapt the availability of macronutrients to maximize plant physiological mineral efficiency per yield (Hawkesford, 2012).

Greenhouse cultivation and indoor growing modules are now a popular alternative to open field cultivations when it comes to early and intensive production. Combined with soilless systems (e.g., hydroponics, aeroponics, etc.), a protected, controlled environment can secure an all-year-round production, hence satisfying the rising demand for high-value fresh leafy vegetables. The environmental and economic costs of the use of non-renewable chemical fertilizers are encouraging the development of more sustainable strategies for mineral biofortification, going beyond the approach of fertilizing crops above the level required for normal growth (Withers and Lord, 2002). In a continuation of our previous paper concerning the bioactive profile (El-Nakhel et al., 2020), in this work, we addressed the possibility of obtaining a higher nutrient efficiency and mineral concentration in the edible part of a crop by concurrently modulating the concentration of three important dietary macrocations, while monitoring the effects on the food quality (Grusak and Dellapenna, 1999; White and Brown, 2010). The work was carried out on lettuce (*Lactuca sativa* L.) because this species is arguably the most important fresh vegetable worldwide and it is almost exclusively eaten raw, thus representing a highly popular low-calorie dietary source of several minerals, fiber, and bioactive compounds (Kim et al., 2016). Lettuce is also a typical crop bred to be used with high nutritional and water input mainly because of its short cycle and high harvest index. Many studies have dealt with the lettuce response to limiting water and nitrogen supply (Seginer, 2003; Kirnak et al., 2016; Malejane et al., 2018). From the nutritional point of view, different reports have focused on the variation in fertilization levels mainly for the nonessential nutrient elements, such as selenium and iodine (Blasco et al., 2008; Ramos et al., 2010; Smoleń et al., 2014). In this work, we investigated the effects of the combinatorial variation of macrocations availability in two morphologically similar butterhead lettuce varieties that strongly differ in leaf color, green or red. *L. sativa* has a remarkable morphological diversity, and cultivated varieties are classified in different horticultural groups, with the butterhead type being one of the most popular (Ryder, 1979). Our aim was to understand the morpho-anatomical, physiological, and biochemical adaptations as well as the efficiency in utilizing mineral resources considering the intraspecific variation in lettuce, the availability of three isosmotic ratios of three macrocations (K, Mg, and Ca), and their interaction in controlled conditions.

## Materials and Methods

### Plant Material and Experimental Setting

Two butterhead lettuce (*L. sativa* L. var. capitata) cultivars, “Descartes” (hereinafter “Green Salanova,” GS) and “Klee” (hereinafter “Red Salanova,” RS) from Rijk Zwaan Italia (Bologna, Italy), were grown in rockwool cubes (7 cm side) using a Nutrient Film Technique (NFT) soilless system based on 2-m long and 8-cm deep closed polypropylene gullies, with a density of 15.5 plants per square meter. Uniform-sized plantlets were transplanted 15 days after sowing (in vermiculite) and harvested 19 days later. We used three nutrient solutions (NSs), each differing only in the cationic proportions of K, Mg, and Ca. Specifically, the macrocation ratio for the NS with a high proportion of potassium (named “SK”) was 0.68 K/0.16 Ca/0.16 Mg, for the NS with a high proportion of Ca (“SCa”) was 0.16 K/0.68 Ca/0.16 Mg, and for the NS with the high proportion of Mg (“SMg”) was 0.16 K/0.16 Ca/0.68 Mg. The concentration (mM) of the three macrocations in the SK were 8.11 K, 0.97 Ca, and 0.97 Mg; in the SCa 1.94 K, 4.06 Ca, and 0.97 Mg; and in the SMg 1.94 K, 0.97 Ca, and 4.06 Mg. The concentration of the other cations and anions was that of a modified Hoagland solution and is reported elsewhere (El-Nakhel et al., 2020). Plants grew in a 28 m^2^ open-air growth chamber (Spagnol, Treviso, Italy) at Department of Agricultural Sciences, University of Naples Federico II (Italy) with a light intensity of 420 μmol/m^2^/s, a photoperiod of 12:12 h, temperature of 24°C (18°C), and relative humidity (RH) of 65% (75%) during the light (dark) period. Treatments were arranged in a randomized complete block design with three replicates, for a total of 18 experimental units (two cultivars × three NS × three replicates), each made of 12 plants in a single NFT gully. Data were recorded excluding the first and the last two plants, considered guards, leaving five plants for the morpho-physiological measurements and three plants, jointly processed, for the compositional analyses.

### Determination of Ionic Composition

Fresh weight (FW) and DW of the leaves were assessed as already described (El-Nakhel et al., 2020). Leaf tissue (three replicates per conditions) was oven-dried at 70°C for 72 h and used for the determination of mineral composition by ion chromatography, using the previously published procedures (El-Nakhel et al., 2020). The total above ground amount of a mineral per plant at maturity (potassium: KTA, calcium: CaTA; magnesium: MgTA) was calculated as reported (Corrado et al., 2020a). The plant science literature presents a significant variety of indices and metrics to measure the utilization efficiency (UtE) of mineral elements. Considering that efficiency is intended as an output-to-input ratio, we calculated the nutrient (K, Mg, and Ca) UtE as a dimensionless ratio of dry biomass (grams per plant) to mineral content (grams per plant) (George et al., 2002; Yang et al., 2003; Hell and Mendel, 2010), hence: KUtE = DW/KTA; MgUtE = DW/MgTA; CaUtE = DW/CaTA. This is equivalent to the physiological mineral efficiency of the lettuce head (Kerbiriou et al., 2014). To cross-compare and summarize the plant response to the variation of the three macroelements, we developed a dimensionless composite indicator of mineral UtE, called normalized average mineral UtE (NAMUtE). Efficiency data of each mineral were first linearly normalized (Z-score) to reduce the measurements to a standard scale (i.e., with 0 mean and SD of 1) and then aggregated using the arithmetic mean, without assigning a weight to each mineral. Finally, to assess the ability of the plant to accumulate mineral nutrients per yield, we calculated the mineral utilization index (KUtI, MgUtI, and CaUtI) as the ratio between the micromoles of minerals and fresh yield (in g) per plant.

### Leaf Anatomical Functional Traits

At harvest, mature leaves were sampled from five plants per treatment, fixed in a 38% formaldehyde, glacial acetic acid, 50% ethanol solution (5:5:90 by volume) and prepared for microscopy according to standard protocols (De Micco et al., 2011; Carillo et al., 2020). In brief, subsamples of leaves were dehydrated and embedded in the acrylic resin JB4 (Polysciences, Hirschberg, Germany) and thin sectioned (5-μm thick) through a rotary microtome. The cross sections, stained with 0.5% Toluidine blue in water, were observed through a transmitted light microscope (BX60; Olympus, Hamburg, Germany). Digital images were captured (Camedia C4040; Olympus, Hamburg, Germany) and subjected to digital image analysis (AnalySIS 3.2; Olympus, Hamburg, Germany) to quantify the following traits: the thickness of leaf lamina, palisade, and spongy parenchyma, the quantity of intercellular spaces in the palisade parenchyma (in six regions per section). Another set of subsamples of leaves was devoted to peeling, and microphotographs of epidermal strips of the upper and the lower lamina surfaces were analyzed to quantify the following stomata traits: stomata frequency (number of stomata per mm^2^ in three regions per peel) and stomata size (length and width of the guard cells in 10 cells per lower epidermis peel).

### Analysis of the Leaf Gas Exchange

Measurements were carried out inside the growth chamber in the above-described environmental conditions. Net photosynthetic CO_2_ assimilation rate (ACO_2_), stomatal resistance (r_s_), and leaf transpiration rate (E) were determined before the harvest on fully expanded leaves (third leaf from the top) whit a portable gas exchange analyzer (LCA-4; ADC BioScientific Ltd., Hoddesdon, UK) fitted with a broad leaf chamber head (window area: 6.25 cm^2^). Photosynthetic photon flux density (PPFD), RH, and carbon dioxide (CO_2_) concentrations were set at ambient values (420 ± 15 μmol m^−2^ s^−1^, RH 66 ± 2% and 385 ± 5 ppm, respectively), and the flow rate of air was 400 ml s^−1^. The intrinsic water-use efficiency (WUEi) was computed as the ratio between ACO_2_ and E.

### Semi-quantification of Phenolic Compounds

Phenolics were extracted and analyzed as described (Rocchetti et al., 2017). The annotated phenolic compounds were firstly assigned into classes and subclasses, and then a semi-quantification approach based on standard solutions (methanol/water 80:20, v/v) of pure (>98%) standard compounds was used, as previously reported (Rocchetti et al., 2017). Cyanidin (anthocyanins and related compounds), catechin (flavanols), quercetin (flavonols), luteolin (flavones and other flavonoids), ferulic acid (hydroxycinnamic and hydroxybenzoic acids and other phenolic acids), sesamin (lignans), resveratrol (stilbenes), and tyrosol (tyrosols and other remaining small molecular weight phenolics) were used as a representative of their respective classes. The results were expressed as mg phenolic equivalents/100 g DM.

### Determination of *in vitro* Antioxidant and Enzyme Inhibitory Effects

Metal chelating, phosphomolybdenum, ferric reducing antioxidant power (FRAP), cupric reducing antioxidant capacity (CUPRAC), 2,2-azino-bis (3-ethylbenzothiazoline-6-sulphonic acid) (ABTS), and 2,2-diphenyl-1-picrylhydrazyl (DPPH) were carried out by using the previously published procedures (Senizza B. et al., 2020). The results were then expressed as Trolox equivalents, while for the metal chelating assay as ethylenediaminetetraacetic acid (EDTA) equivalents. In addition, by adopting different assays, we inspected the inhibitory capacity of each extract against acetylcholinesterase [AChE, (type-VI-S, EC 3.1.1.7), butyrylcholinesterase (BChE, EC 3.1.1.8)] (by Ellman's method), α-amylase (EC. 3.2.1.1), α-glucosidase (EC. 3.2.1.20), and tyrosinase (EC. 1.14.18.1). The results were expressed for tyrosinase as equivalents of kojic acid (KAE), for AChE and BChE as galantamine (GALAE), and for α-amylase and α-glucosidase as acarbose (ACAE).

### Statistical Analysis

Data were subjected to statistical analysis by using SPSS 26 software (IBM, Armonk, NY, USA). The main effect of the categorical independent factors (i.e., cultivar, C, and NS) and their interaction on the continuous dependent variables were analyzed through the two-way ANOVA. In case of rejection of the null hypothesis, the Tukey's HSD test was performed (*p* < 0.05). The *post hoc* analysis included one-way ANOVA followed by Tukey's HSD test (for the NS factor, *p* < 0.05) or the Student's *t*-test (for the C factor, *p* < 0.05). The Kolmogorov–Smirnov test was performed to check for normality. Percent data were transformed through arcsine function before statistical analysis. The correlation coefficients between enzymatic assays and different phenolic subclasses were then determined according to Pearson's analysis in SPSS 26 (two-tailed).

## Results

### Effects on the Mineral UtE

The NSs had a large impact on the elemental composition of the lettuce leaves. For instance, the relationship between the dry (or fresh) biomass output at harvest and the mineral amount displayed a significant variation considering the cultivar, the different ratios of macrocations in the NS, and their interaction (Table 1). As expected, the different concentrations of K, Mg, and Ca considerably affected the plant's mineral utilization. Overall, a higher amount of macrocation is associated with lower physiological efficiency in terms of produced biomass. Nonetheless, the multifactorial variation of the macrocations indicated that the UtE is not a linear function of the concentration in the NS, as implied by the rank of the different UtE values for each NS. The two genotypes did not statistically differ in the utilization of K and Mg according to the ratio of the macrocations. The interaction of factors was also significant for the efficiency of the plant, with the utilization of Mg most highly affected by the different nutrient regimes and its interaction with the genotypes. The main effect of the genotype over the UtE of the macrocations under investigation was reduced while the interaction genotype × NS was more significant for Mg, followed by Ca and K. To account for the different magnitude of variations of K, Ca, and Mg in plants, we calculated a NAMUtE by aggregating standardized values. The mean UtE was not statistically affected by the NS, indirectly supporting the notion that the observed variations for each NSs are due to the concurrent alterations in mineral availability (removed in the aggregated analysis). In contrast, the RS had a slightly higher normalized UtE than the GS, indicating a genotype-dependent higher efficiency in the utilization of macrocations.

**Table 1:** Effects of the cultivar (C) and of the nutrient solution (NS) on the utilization of K, Mg, and Ca in leaves.

**Source of variance**	**KUtE (g DW/g K)**	**CaUtE (g DW/g Ca)**	**MgUtE (g DW/g Mg)**	**NAMUtE**	**KUtI (μmol K/g FW)**	**CaUtI (μmol Ca/g FW)**	**MgUtI (μmol Mg/g FW)**
**Cultivar (C)**
GS	19.01 ± 5.10	143.63 ± 72.90	322.80 ± 150.40	−0.13 ± 0.17b	66.87 ± 18.36	10.45 ± 5.78	8.18 ± 5.64
RS	19.34 ± 6.39	194.48 ± 77.47	335.03 ± 195.58	0.13 ± 0.15a	67.28 ± 23.76	7.26 ± 4.04	8.21 ± 5.45
**Nutrient solution (NS)**
SCa	24.12 ± 0.97a	78.48 ± 16.79c	331.55 ± 36.79b	−0.09 ± 0.07	49.28 ± 1.46c	15.30 ± 3.03a	5.83 ± 0.71c
SK	11.71 ± 0.79c	184.66 ± 49.59b	523.96 ± 59.03a	0.01 ± 0.29	94.61 ± 4.92a	6.24 ± 1.81b	3.43 ± 0.45b
SMg	21.69 ± 1.32b	244.02 ± 28.51a	131.23 ± 11.67c	0.08 ± 0.19	57.34 ± 3.52b	5.04 ± 0.8c	15.32 ± 1.65a
**C** **×** **NS**
GS × SCa	23.29 ± 0.27ab	63.79 ± 3.28c	363.64 ± 13.12c	−0.14 ± 0.02bc	50.39 ± 1.10de	17.98 ± 0.93a	5.19 ± 0.16
GS × SK	12.36 ± 0.32c	140.11 ± 11.69b	471.74 ± 15.35b	−0.25 ± 0.07c	90.6 ± 0.87b	7.83 ± 0.71c	3.82 ± 0.19
GS × SMg	21.38 ± 1.36b	226.98 ± 33.29a	133.03 ± 16.67e	−0.01 ± 0.25abc	59.61 ± 3.91c	5.54 ± 0.82d	15.54 ± 1.99
RS × SCa	24.95 ± 0.45a	93.17 ± 6.83c	299.46 ± 11.05d	−0.04 ± 0.08abc	48.16 ± 0.63e	12.62 ± 0.72b	6.46 ± 0.15
RS × SK	11.06 ± 0.44c	229.22 ± 7.42a	576.19 ± 17.13a	0.26 ± 0.07a	98.62 ± 3.42a	4.64 ± 0.20d	3.04 ± 0.14
RS × SMg	22.00 ± 1.48b	261.06 ± 7.29a	129.43 ± 7.25e	0.17 ± 0.06ab	55.06 ± 0.44cd	4.53 ± 0.41d	15.11 ± 1.66
**Significance**
C	ns	***	ns	***	ns	***	ns
NS	***	***	***	ns	***	***	***
C × NS	*	**	***	*	**	***	ns

*KUtE, MgUtE, and CaUtE are the K, Mg, and Ca utilization efficiency (UtE), respectively. NAMUtE is the normalized average mineral UtE. KUtI, CaUtI, and MgUtI are the K, Mg, and Ca utilization index, respectively. Data are expressed as mean ± SE*.

ns, *, **, *** Nonsignificant or significant at p <0.05, p <0.01, and p <0.001, respectively. Different letters within each column indicate significant differences according to Tukey's HSD test (p <0.05). The factor “C” was compared with the independent Student's t-test.

To assess the effects on the nutritional value of lettuce, we evaluated the ability to accumulate minerals per yield at maturity. Not surprisingly, the NS factor always had a significant impact and, in molar terms, the dominant cation in the NS was present in at least twice the amount of the other two macronutrients. Moreover, significant mineral interactions were also evident. For each NS, the molar quantity in leaves of the two non-dominant macrocations (for instance, Mg and Ca for the SK solutions) varies according to the NSs (i.e., according to the type of dominant macrocation), although we used isosmotic NSs with fixed molar ratios (e.g., the quantity of Mg is the same in the SK and SCa solutions). Differences among the three macroelements were present considering the effect of the genotype. Only the utilization index of calcium was significantly affected by the genotype, and the interaction of factors did not influence only the MgUtI. Overall, the data revealed the distinctive accumulations of the macrocations in leaves and indicated that mineral interaction specifically influences the mineral utilization index of lettuce.

### Effects on the Leaf Anatomy

Microscopy observations of the leaf lamina showed that the RS had thinner leaves. Their mesophyll is characterized by a higher incidence of palisade parenchyma made of poorly elongated cells (Table 2 and Supplementary Figure 1). The C × NS interaction had a significant effect on all mesophyll parameters but spongy parenchyma thickness, leading to the highest ratio palisade/spongy parenchyma in RS treated with the SK solution. In both cultivars, the SMg solution induced more compact spongy parenchyma as indicated by the significantly lower percent of intercellular spaces (Table 2). Regarding stomatal traits, only the width of guard cells was significantly affected by the cultivar as the main factor (Table 3). Stomatal frequency was influenced differently at the upper and lower epidermis by the composition of the NS, indicating a different control of epidermal cell differentiation at the two lamina sides. SK significantly reduced and increased stomata frequency at the upper and the lower epidermis, respectively (Table 3). The C × NS interaction had a significant effect on the stomatal frequency and size of guard cells at the lower epidermis, with the higher occurrence of smaller stomata in RS treated with SK (Table 3), compared to the other treatments. On the contrary, a lower frequency of larger stomata was observed in GS treated with SCa compared to the other treatments, which showed intermediate values (Table 3).

**Table 2:** Effects of the C and of the NS on mesophyll traits.

**Source of variance**	**Leaf lamina thickness (μm)**	**Palisade parenchyma thickness**	**Spongy parenchyma thickness**	**Palisade/spongy ratio**	**Intercellular spaces (%)**
		**(μm)**	**(μm)**		
**Cultivar (C)**
GS	324.1 ± 8.33	39.60 ± 1.25	252.3 ± 7.33	0.159 ± 0.01	58.72 ± 2.78
RS	279.7 ± 4.90	44.47 ± 2.19	203.7 ± 4.83	0.222 ± 0.01	58.44 ± 3.24
**Nutrient solution (NS)**
SCa	305.1 ± 14.6	40.83 ± 1.11b	248.0 ± 12.4a	0.169 ± 0.01b	63.62 ± 1.68a
SK	296.7 ± 12.1	49.61 ± 1.98a	209.6 ± 8.44b	0.242 ± 0.02a	67.77 ± 1.45a
SMg	303.8 ± 4.25	35.68 ± 0.78c	226.4 ± 8.42b	0.159 ± 0.01b	44.35 ± 1.73b
**C** **×** **NS**
GS × SCa	339.9 ± 16.4a	39.15 ± 0.96bcd	280.9 ± 9.69	0.140 ± 0.00c	60.17 ± 1.98b
GS × SK	323.2 ± 16.8a	44.64 ± 1.39b	231.7 ± 7.89	0.193 ± 0.01b	69.50 ± 1.97a
GS × SMg	309.2 ± 7.58a*b*	35.01 ± 1.35d	244.3 ± 9.02	0.144 ± 0.01bc	46.48 ± 2.50c
RS × SCa	270.3 ± 9.15b	42.50 ± 1.80bc	215.0 ± 7.06	0.199 ± 0.01b	67.08 ± 1.66ab
RS × SK	270.3 ± 5.19c	54.58 ± 1.83a	187.5 ± 3.74	0.291 ± 0.01a	66.03 ± 2.01ab
RS × SMg	298.5 ± 3.10a*b*	36.34 ± 0.84cd	208.5 ± 8.82	0.175 ± 0.00b	42.23 ± 2.22c
**Significance**
C	***	ns	***	***	ns
NS	ns	***	***	***	***
C × NS	*	*	ns	***	*

*Data are expressed as mean ± SE*.

ns, *, *** Non-significant or significant at p <0.05, p <0.01, and p <0.001, respectively. Different letters within each column indicate significant differences according to Tukey's HSD test (p <0.05). The factor “C” was compared with the independent Student's t-test.

**Table 3:** Effects of the C and of the NS on leaf stomatal parameters.

**Source of variance**	**Stomata frequency upper epidermis**	**Stomata frequency lower epidermis**	**Guard cell length**	**Guard cell width**
	**(no. mm^**−2**^)**	**(no. mm^**−2**^)**	**(μm)**	**(μm)**
**Cultivar (C)**
GS	79.72 ± 4.10	74.31 ± 1.68	32.47 ± 0.60	12.15 ± 0.15
RS	69.84 ± 3.14	74.01 ± 2.65	32.17 ± 0.60	11.50 ± 0.18
**Nutrient solution (NS)**
SCa	71.64 ± 2.45b	72.34 ± 1.73b	32.61 ± 0.93	11.88 ± 0.33a
SK	61.65 ± 2.52c	80.19 ± 2.22a	31.67 ± 0.44	11.48 ± 0.12b
SMg	91.04 ± 2.88a	69.95 ± 2.94b	32.68 ± 0.74	12.13 ± 0.12a
**C** **×** **NS**
GS × SCa	73.33 ± 4.45	68.71 ± 2.15bc	35.03 ± 0.75a	12.79 ± 0.17a
GS × SK	67.02 ± 3.41	76.57 ± 3.15ab	31.74 ± 0.66b	11.73 ± 0.10bc
GS × SMg	98.79 ± 1.89	77.65 ± 1.80ab	30.64 ± 0.46b	11.93 ± 0.17bc
RS × SCa	69.95 ± 2.40	75.97 ± 1.50ab	30.19 ± 0.65b	10.96 ± 0.21d
RS × SK	56.27 ± 1.56	83.81 ± 2.37a	31.60 ± 0.65b	11.23 ± 0.16cd
RS × SMg	83.30 ± 1.95	62.24 ± 2.46c	34.71 ± 0.44a	12.32 ± 0.13b
**Significance**
C	ns	ns	ns	**
NS	***	***	ns	**
C × NS	ns	***	***	***

*Data are expressed as mean ± SE*.

ns, **, *** Nonsignificant or significant at p < 0.01, and p < 0.001, respectively. Different letters within each column indicate significant differences according to the Tukey's HSD test (p < 0.05). The factor “C” was compared with the independent Student's t-test.

### Effects on Leaf Gas Exchanges

The RS had higher leaf photosynthetic rates, while the factor “C” did not influence stomatal resistance and transpiration (Table 4). The intrinsic water efficiency was also affected only by the genotype factor. The ratio of macrocations had a significant effect on ACO_2_, RS, and E. The best photosynthetic performance was obtained by using SK, although a lower stomatal resistance was associated with increased evapotranspiration, leading to an alteration in the efficiency in the water use. Moreover, with the SK solution, there was a significant interaction with the genotype on the analyzed parameters mainly because of the improved performance of the SK of the red variety.

**Table 4:** Effects of the C and of the NS on chlorophyll and gas exchange parameters of the leaves.

**Source of variance**	**A_**CO2**_**	**r_**s**_**	**E**	**WUEi**
	**(μmol CO_**2**_ m^**−2**^ s^**−1**^)**	**(m^**2**^ s^**−1**^ mol^**−1**^)**	**(mol H_**2**_O m^**−2**^ s^**−1**^)**	**(μmol CO_**2**_ mol^**−1**^ H_**2**_O)**
**Cultivar (C)**
GS	8.55 ± 0.22	4.43 ± 0.39	2.62 ± 0.09	3.36 ± 0.17
RS	13.02 ± 0.31	4.42 ± 0.22	2.60 ± 0.07	5.03 ± 0.09
**Nutrient solution (NS)**
SCa	10.19 ± 0.56b	5.28 ± 0.44a	2.35 ± 0.10c	4.43 ± 0.29
SK	11.69 ± 0.76a	3.55 ± 0.25b	2.87 ± 0.07a	4.08 ± 0.25
SMg	10.47 ± 0.71b	4.45 ± 0.31ab	2.61 ± 0.07b	4.08 ± 0.31
**C** **×** **NS**
GS × SCa	8.42 ± 0.24c	5.37 ± 0.84	2.37 ± 0.19	3.72 ± 0.38
GS × SK	9.18 ± 0.45c	3.54 ± 0.46	2.82 ± 0.13	3.28 ± 0.19
GS × SMg	8.05 ± 0.32c	4.39 ± 0.60	2.67 ± 0.13	3.09 ± 0.28
RS × SCa	11.96 ± 0.51b	5.19 ± 0.38	2.34 ± 0.08	5.14 ± 0.20
RS × SK	14.21 ± 0.42a	3.55 ± 0.24	2.91 ± 0.07	4.89 ± 0.14
RS × SMg	12.89 ± 0.34b	4.51 ± 0.25	2.55 ± 0.08	5.08 ± 0.13
**Significance**
C	***	ns	ns	***
NS	***	*	***	ns
C × NS	*	ns	ns	ns

*ACO_2_, net photosynthetic CO_2_ assimilation rate; r_s_, stomatal resistance; E, leaf transpiration rate; WUEi, intrinsic water-use efficiency. Data are expressed as mean ± SE*.

ns, *, *** Nonsignificant or significant at p < 0.05, and p < 0.001, respectively. Different letters within each column indicate significant differences according to the Tukey's HSD test (p < 0.05). The factor “C” was compared with the independent Student's t-test.

### Effects on Phenolic Compounds

Analyzing the variation in phenolic compounds using an untargeted approach based on UHPLC-QTOF mass spectrometry, we putatively annotated 342 compounds, including 174 flavonoids, 74 phenolic acids, 19 lignans, and 67 low molecular weight compounds (Supplementary Table 1). The RS showed a higher quantity of total polyphenols. The content of lignans (1,025 mg/100 g), tyrosols and low molecular weight phenolics (1,009 mg/100 g), phenolic acids (671.6 mg/100 g), and flavones (232.5 mg/100 g) equivalents in RS was higher compared to the GS, possessing values of 667.1 mg/100 g, 725 mg/100 g, 363.4 mg/100 g, and 205 mg/100 g, respectively. The less abundant phenolic classes were cyanidin equivalents and related compounds, flavanols and stilbenes, with concentrations ranging from 29 to 51 mg/100 g. The different ratios of K, Mg, and Ca in the NS significantly affected the phenolic composition of the varieties. In particular, the SCa in RS increased flavanols, flavones, tyrosols, and phenolic acids (*p* < 0.05). Conversely, an accumulation of flavones and tyrosols (222.7 and 834.4 mg/100 g, respectively), was observed in the GS variety with the SK. In both cultivars, the accumulation of flavonols was promoted by a high concentration of Mg, being 54.55 mg/100 g for GS and 91.53 mg/100 g for RS.

The annotated phenolic compounds (classified according to a representative standard per class/subclass) were quantified and expressed as mg/100 g equivalents (Table 5). On one hand, we found an abundance of tetramethylscutallarein, 8-prenylnaringenin, 6-geranylnaringenin (flavones), 7-oxomatairesinol, isohydroxymatairesinol, and dimethylmatairesinol (lignans), together with ferulic and hydroxycaffeic acids, when considering GS. On the other hand, the compounds characterizing the RS were the flavones dihydroxyflavone and sinensetin, the lignans conidendrin and pinoresinol, together with the hydroxycinnamic acids hydroxycaffeic acid and 24-methyllathosterol ferulate.

**Table 5:** Effects of the C and of the NS on the untargeted phenolic profile of phenolic compounds of the leaves.

	**Cyanidin eq. (mg/100 g DW)**	**Catechin eq. (mg/100 g DW)**	**Luteolin eq. (mg/100 g DW)**	**Quercetin eq. (mg/100 g DW)**	**Sesamin eq. (mg/100 g DW)**	**Tyrosol eq. (mg/100 g DW)**	**Ferulic eq. (mg/100 g DW)**	**Resveratrol eq. (mg/100 g DW)**
**Cultivar (C)**
GS	25.18 ± 0.59	22.29 ± 0.94	205.9 ± 8.84	53.66 ± 3.10	667.1 ± 21.6	725.8 ± 26.41	363.4 ± 6.87	33.42 ± 1.98
RS	29.78 ± 0.89	29.51 ± 1.79	232.5 ± 12.5	84.99 ± 2.74	1,025 ± 50.0	1,009 ± 39.43	671.6 ± 16.55	50.78 ± 6.25
**Nutrient solution (NS)**
SCa	29.38 ± 1.06a	31.55 ± 2.37a	220.3 ± 18.8	69.74 ± 5.25	899.0 ± 85.9	946.0 ± 73.91a	537.3 ± 58.37	53.36 ± 7.21a
SK	25.38 ± 0.85b	25.47 ± 1.08b	202.7 ± 10.2	65.20 ± 6.16	862.1 ± 50.1	883.6 ± 39.33a	502.5 ± 41.27	33.94 ± 2.75b
SMg	27.67 ± 1.22ab	20.68 ± 0.89c	234.7 ± 9.17	73.04 ± 6.19	777.0 ± 70.8	772.4 ± 47.65b	512.7 ± 45.30	39.01 ± 6.50ab
**C** **×** **NS**
GS × SCa	27.24 ± 0.47	24.44 ± 1.82bc	176.2 ± 13.3b	54.16 ± 1.51	684.4 ± 36.6	710.4 ± 27.16cd	349.6 ± 9.03c	39.25 ± 2.36
GS × SK	23.53 ± 0.53	23.77 ± 0.94bc	222.7 ± 11.0ab	52.27 ± 9.49	719.3 ± 36.3	834.2 ± 39.64bc	370.0 ± 16.85c	25.59 ± 1.55
GS × SMg	24.76 ± 1.29	18.65 ± 0.85c	218.9 ± 15.2ab	54.55 ± 2.26	597.7 ± 23.3	632.6 ± 25.94c	370.6 ± 7.72c	35.44 ± 3.50
RS × SCa	31.53 ± 1.71	38.67 ± 1.11a	264.4 ± 24.5a	85.33 ± 4.67	1,114 ± 113	1,182 ± 33.25 a	725.0 ± 28.64a	67.47 ± 11.99
RS × SK	27.22 ± 1.24	27.16 ± 1.76b	182.7 ± 13.3b	78.12 ± 3.16	1,005 ± 39.5	933.0 ± 65.25b	635.0 ± 13.70b	42.29 ± 1.72
RS × SMg	30.57 ± 1.22	22.70 ± 1.07bc	250.4 ± 6.22a	91.53 ± 5.20	956.3 ± 93.0	912.1 ± 38.81b	654.8 ± 29.86b	42.59 ± 12.98
**Significance**
C	***	***	ns	***	***	***	***	**
NS	**	***	ns	ns	ns	***	ns	*
C × NS	Ns	***	***	ns	ns	***	*	ns

*Data are expressed as semiquantitative values (mean ± SE). Cyanidin was used for anthocyanins and related compounds, catechin for flavanols, quercetin for flavonols, luteolin for flavones and remaining flavonoids, sesamin for lignans, ferulic acid for phenolic acids (hydroxycinnamics and phenolic acids), resveratrol for stilbenes, and tyrosol for tyrosols and other low molecular weight phenolics*.

ns, *, **, *** Non-significant or significant at p <0.05, p <0.01, and p <0.001, respectively. Different letters within each column indicate significant differences according to the Tukey's HSD test (p <0.05). The factor “C” was compared with the independent Student's t-test.

### Effects on the *in vitro* Antioxidant Capacity

The antioxidant capacity of the leaf extracts was evaluated *in vitro* in different aspects, namely as radical scavenging (DPPH and ABTS), reducing power (FRAP and CUPRAC), total antioxidant capacity (phosphomolybdenum), and metal chelating ability. Overall, looking at GS, the higher DPPH scavenging properties and phosphomolybdenum activities were found for the extracts with a higher portion of Mg, resulting in 4.53 mg TE/g and 0.80 mmol TE, while the SK was the NS that better promoted the cupric reduction capacity (29.83 mg TE/g) and metal chelating ability (21.90 mg EDTAE/g) (Table 6). A lower antioxidant capacity characterized the SCa extracts. Similarly, for the RS, the highest ABTS scavenging potential (28.07 mg TE/g) and reducing power were present in the SK leaf extracts. Cupric reduction capacity and ferric reduction activity recorded the values of 53.79 mg TE/g and 24.04 mg TE/g, respectively. The weakest scavenger was found to be the SMg extract (ABTS = 18.59 and DPPH = 10.00 mg TE/g) (Table 6). When comparing the two varieties, RS exhibited a stronger and significant antioxidant potential, likely due to the presence of higher values of total phenolic content (TPC) and total flavonoid content (TFC). Finally, a strong correlation (*p* < 0.01) was found between TPC and ABTS (0.640), CUPRAC (0.811), FRAP (0.77), and metal chelating (0.846).

**Table 6:** Effects of the C and of the NS of antioxidant capacity of the leaves.

**Source of variance**	**DPPH**	**ABTS**	**CUPRAC**	**FRAP**	**Phosphomolydenum**	**Metal chelating**
	**(mg TE)**	**(mg TE)**	**(mg TE)**	**(mg TE)**	**(mmol TE)**	**(mg EDTA)**
**Cultivar (C)**
GS	3.13 ± 0.28	11.68 ± 0.88	26.38 ± 0.74	13.32 ± 0.52	0.76 ± 0.02	15.01 ± 1.34
RS	12.14 ± 1.12	22.86 ± 1.33	49.30 ± 1.67	20.50 ± 0.83	0.95 ± 0.03	17.97 ± 0.93
**Nutrient solution (NS)**
SCa	9.44 ± 2.37a	15.27 ± 2.32b	36.24 ± 3.88b	15.47 ± 1.36b	0.87 ± 0.06	11.03 ± 0.77c
SK	6.20 ± 1.30b	21.49 ± 2.23a	41.81 ± 3.92a	19.82 ± 1.44a	0.84 ± 0.03	22.08 ± 0.15a
SMg	7.26 ± 0.93ab	15.05 ± 1.28b	35.46 ± 3.32b	15.44 ± 0.79b	0.86 ± 0.03	16.36 ± 0.75b
**C** **×** **NS**
GS × SCa	2.37 ± 0.09c	8.62 ± 0.32	24.41 ± 1.19	11.21 ± 0.38e	0.71 ± 0.02c	8.75 ± 0.38d
GS × SK	2.48 ± 0.32c	14.90 ± 1.74	29.83 ± 0.64	15.60 ± 0.83cd	0.77 ± 0.03bc	21.90 ± 0.11a
GS × SMg	4.53 ± 0.30c	11.51 ± 0.88	24.88 ± 0.33	13.16 ± 0.09de	0.80 ± 0.03bc	14.38 ± 0.80c
RS × SCa	16.51 ± 2.16a	21.92 ± 2.43	48.07 ± 2.97	19.73 ± 0.87b	1.03 ± 0.06a	13.30 ± 0.65c
RS × SK	9.93 ± 1.34b	28.07 ± 1.20	53.79 ± 3.12	24.04 ± 1.17a	0.91 ± 0.03b	22.27 ± 0.27a
RS × SMg	10.00 ± 0.85b	18.59 ± 1.19	46.04 ± 1.90	17.72 ± 0.82bc	0.92 ± 0.04b	18.35 ± 0.49b
**Significance**
C	***	***	***	***	***	ns
NS	*	***	**	***	ns	***
C × NS	**	ns	ns	*	*	***

*Data are expressed as mean ± SE*.

ns, *, **, *** Nonsignificant or significant at p <0.05, p <0.01, and p <0.001, respectively. Different letters within each column indicate significant differences according to the Tukey's HSD test (p <0.05). The factor “C” was compared with the independent Student's t-test.

### *In vitro* Inhibitory Activity on Selected Enzymes

To evaluate the effects of the ratios of macrocations on the functional lettuce properties, we detected the inhibition of the leaf extracts on selected enzymatic activities. The strongest BChE inhibition was observed in SK solution (5.71 mg GALAE/g), followed by the SMg (4.39 mg GALAE/g) and SCa (4.00 mg GALEA/g) for GS. For the RS, the SCa demonstrated a higher inhibition activity (4.10 mg GALAE/g) than the SMg (3.90 mg GALAE/g) (Table 7). Also, lower inhibition properties were found in the GS against acetylcholinesterase, resulting in the values of 2.17 and 2.31 mg GALAE/g from SMg and SK, respectively. No inhibitory potential was revealed by SCa. With respect to the RS, the most effective AChE inhibitor was SCa, recording activities of 2.51 mg GALAE/g. Conversely, SK demonstrated the weakest inhibition potential with 1.25 mg GALAE/g. A significant correlation between glucosidase (*p* < 0.001) and acetylcholinesterase (*p* < 0.05) and the NS was found, instead of BChe and tyrosinase, where no significant correlation was detected (Table 7).

**Table 7:** Effects of the C and of the NS of the enzymatic inhibitory properties of the leaf extract.

**Source of variance**	**AChE**	**BChE**	**Tyrosinase**	**Amylase**	**Glucosidase**
	**(mg GALAE)**	**(mg GALAE)**	**(mg KAE)**	**(mmol ACAE)**	**(mmol ACAE)**
**Cultivar (C)**
GS	2.26 ± 0.05	4.70 ± 0.23	55.69 ± 1.21	0.26 ± 0.00	0.76 ± 0.01
RS	1.94 ± 0.24	4.27 ± 0.21	60.13 ± 1.29	0.30 ± 0.01	0.74 ± 0.02
**Nutrient solution (NS)**
SCa	2.51 ± 0.16a	4.05 ± 0.19b	53.54 ± 1.36c	0.27 ± 0.01b	0.71 ± 0.02b
SK	1.95 ± 0.23b	5.24 ± 0.26a	63.18 ± 1.24a	0.30 ± 0.01a	0.76 ± 0.02a
SMg	2.11 ± 0.07b	4.15 ± 0.22b	57.01 ± 0.94b	0.27 ± 0.01b	0.78 ± 0.01a
**C** **×** **NS**
GS × SCa		4.00 ± 0.22	50.14 ± 0.63	0.25 ± 0.00c	0.76 ± 0.01a
GS × SK	2.31 ± 0.07ab	5.71 ± 0.30	60.28 ± 0.68	0.27 ± 0.01bc	0.73 ± 0.02a
GS × SMg	2.17 ± 0.01ab	4.39 ± 0.24	56.64 ± 1.89	0.26 ± 0.01bc	0.79 ± 0.00a
RS × SCa	2.51 ± 0.16a	4.10 ± 0.33	56.93 ± 1.78	0.29 ± 0.01b	0.66 ± 0.02b
RS × SK	1.25 ± 0.03c	4.78 ± 0.34	66.08 ± 1.71	0.32 ± 0.01a	0.79 ± 0.02a
RS × SMg	2.06 ± 0.16b	3.92 ± 0.37	57.39 ± 0.52	0.27 ± 0.01bc	0.77 ± 0.02a
**Significance**
C	ns	ns	*	***	ns
NS	**	**	***	***	***
C × NS	*	ns	ns	*	***

*Data are expressed as mean ± SE*.

ns, *, **, *** Nonsignificant or significant at p <0.05, p <0.01, and p <0.001, respectively. Different letters within each column indicate significant differences according to the Tukey's HSD test (p <0.05). The factor “C” was compared with the independent Student's t-test.

### Correlation Analysis

To highlight the phenolic contribution to the inhibitory and antioxidant activities of the two lettuce varieties, we measured the linear correlation between the two data sets (Supplementary Table 2). Strong correlations (*p* < 0.01) were found in GS between TPC and FRAP (0.77), CUPRAC (0.81), and metal chelating (0.84), similarly to TFC, resulting in the values of 0.82, 0.73, and 0.84, respectively. Significant correlations were not present with the investigated classes of polyphenols, but a correlation between tyrosols (*p* < 0.05) and ABTS (0.50), CUPRAC (0.52), and metal chelating (0.51) was observed. A negative correlation (*p* < 0.01) existed between cyanidin equivalents and radical scavenging capacities (ABTS= −0.63), reducing powers (FRAP= −0.59), and metal chelating (−0.67). Moreover, stilbenes presented a strong (*p* < 0.01) non-positive correlation with ABTS, CUPRAC, and FRAP, which is 0.67, −0.62, and −0.67, respectively. Regarding the inhibitory activities, flavones and lignans demonstrated a major contribution (*p* < 0.05) in amylase inhibition, while flavonols showed activity against glucosidase.

Tyrosols, phenolic acids, and stilbenes were the main contributors to radical scavenging (DPPH) and total antioxidant capacity in RS. We observed no positive correlation between flavanols and glucosidase inhibitions (−0.59) and flavones and tyrosinase (−0.59). Also, the data analysis revealed a correlation (*p* < 0.05) between AChE assay and lignans (0.54), but no other significant contributors to cholinesterase inhibitions were found. TFC and TPC better explained both antioxidants and inhibitory activities, thus demonstrating strong correlations with most of the assays done.

## Discussion

We investigated the range of effects that the concurrent variations of three macrocations (Ca, Mg, and K) in two lettuce varieties with different leaf colors. Our focus was on the elemental composition and the morphological and functional traits of the edible product. The UtE of the three macrocations was inversely correlated to their amount in the NS, irrespective of the plant variety, indicating that the ability to produce higher biomass per unit of mineral applied increases at low input conditions. However, the UtE was not linearly dependent on the concentrations of macrocations because significant interactions were present among Ca, Mg, and K. For instance, the CaUtE was higher for the SMg solution while MgUtE had the maximum value for the SK. In addition to the interaction of cations, it is noteworthy that clear differences were present also considering the effect of the variety, and the interaction of factors. Calcium was affected by all these factors and its UtE displayed higher relative variations. In lettuce leaves, calcium concentration was also influenced by the fertilization of non-essential elements (i.e., iodine or selenium), indicating that this macroelement is probably very sensitive to variation to mineral nutrition (Smoleń et al., 2014). Moreover, the two lettuce varieties did not differ for the K and Mg efficiency but responded differently to the different isosmotic variations, further indicating a specific plant adaptation to the heterogeneous mineral availability. Considering the different absolute amounts of Ca, Mg, and K in leaves, we used a standardized score to evaluate the overall macrocation UtE. According to this composite indicator, a genotypic difference was evident, with the RS having a statistically higher value, while differences among NSs were very small. Although the concentration of the analyzed mineral elements was well above those expected to give deficiency symptoms, previous works indicated that isosmotic macrocation variation also influences yield (Fallovo et al., 2009; Corrado et al., 2021). Therefore, the higher nutrient UtE (in terms of dry biomass production) of a plant was also evaluated considering the fresh mass of the edible products. Under these perspectives, our work indicated that the two varieties did not differ in the utilization index, while higher amounts of the mineral element in the NS are associated with higher mineral content per FW. The observed inverse correlation between mineral efficiency (in terms of DM return) and accumulation (in terms of concentration per FW) indicated a trade-off with obvious dietary and agronomically significant implications. The genetic base of this phenomenon may be complex (Zhang et al., 2020), since the concentration of macrocations in plants is considered more tightly regulated than that of trace elements because of the need to guarantee and maintain a suitable optimal range (Yang et al., 2018).

The suboptimal availability of mineral nutrients impaired plant growth and photosynthesis at different levels. K and Mg crucially contribute to photosynthesis and long-distance photosynthate translocation (Tränkner et al., 2018). Both K and Mg play common roles in photosynthesis and photoassimilate translocation. They are essential for the synthesis of photosynthetic protein, the development of a well-structured organization of grana and stroma lamellae, and the regulation of the source-sink translocation of photoassimilates (Tränkner et al., 2018). In addition, Mg is required for chlorophyll synthesis while K is important for the direct control of stomata movements and crucial during leaf morphogenesis for its influence on the development of specific leaf anatomical traits linked with mesophyll conductance (Fischer, 1968; Andrés et al., 2014). The best photosynthetic performance was obtained by using the K-rich NS (higher values of ACO_2_ and E, and lower values of r_s_), compared to the other two NSs, most likely because of a more efficient stomatal regulation (Outlaw, 1983; Jákli et al., 2017). Interestingly, the RS cultivar had better performance with the SK solution. In this work, we demonstrated that the SK determined in the RS variety the formation of leaves with a higher frequency of smaller stomata compared to the other conditions. Quicker stomata response is typical of leaves with a higher frequency of smaller stomata compared to leaves with fewer large stomata (provided that pore area is the same), and it is an indicator of greater potential for photosynthesis (Drake et al., 2013; Raven, 2014). In other species too, the plant morphogenetic reaction to suboptimal conditions (besides light) comprised the alteration of stomata traits favoring the formation of small and frequent stomata to maintain high photosynthetic rates also under environmental constraints (Rouphael et al., 2017; Amitrano et al., 2019; Corrado et al., 2020b). K supply also has an effect on leaf parenchyma traits related to mesophyll conductance (Tränkner et al., 2018). It has been recently reported that the total photosynthesis limitation due to reduced mesophyll conductance is increased in K-deficient compared to K-sufficient plants, supporting the idea that K is needed to achieve a good CO_2_ diffusion through the mesophyll (Lu et al., 2016; Jákli et al., 2017). In the RS, the distribution of SK has determined the formation of thinner leaves characterized by a higher incidence of palisade parenchyma and increased volume of intercellular spaces compared to the other conditions. This leaf structure would have reduced the mesophyll resistance to CO_2_, leaving enough space for gaseous CO_2_ diffusion, at the same time leading to a significant increase in the mesophyll resistance to water flow. The reduced cell connections are responsible for a reduced rate of water flux (Buckley et al., 2015; Amitrano et al., 2019). Overall, the analysis of anatomical and ecophysiological traits implies that the RS treated with SK is characterized by more efficient control of gas exchanges, while the GS appeared less sensitive. The relevant genotypic-specific response is consistent with an interconnected and complex feedback loop and interactions affecting single traits (Tränkner et al., 2018).

The genetic factor had a more pronounced effect on phenolic profile. The RS had a higher amount of all the measured classes except for flavones. Lignan and flavonol equivalents were the polyphenols affected by the genotype and, interestingly, these classes are reported to increase significantly in the RS under strongly limiting nutrient conditions (Senizza A. et al., 2020). This further illustrates that the described response to macrocation variation differs from that due to low nutrient availability. Each NS determined a distinctive accumulation of some cyanidin-related compounds, even if concentrations remained low. For instance, SCa primarily determined a major increase of glycosidic forms of pelargonidin (e.g., pelargonidin 3-*O*-glucoside, pelargonidin 3-*O*-rutinoside, and pelargonidin 3-*O*-arabinoside). Although anthocyanin biosynthesis has been investigated in many plants, there is limited knowledge on the effects of different macrocations on anthocyanins profile (El-Nakhel et al., 2020; Senizza B. et al., 2020). The different ratios of macrocations were also able to elicit selected anthocyanins in an NS-specific manner. Concerning flavonols, quercetin 3-*O*-rutinoside was the compound mostly affected by the different macrocations. Nonetheless, SCa induced a higher accumulation of this compound in GS while SMg promoted a stronger accumulation in the RS. Moreover, ferulic acid derivatives were confirmed to be among the most abundant phenolics (Sofo et al., 2016) and the application of SK induced the stronger accumulation of ferulic acid 4-*O*-glucoside only in GS lettuce. Therefore, our data indicated that the genetic background deeply influenced the phenolic composition, and that the ratio of the three macrocations can be exploited for targeted modulation of their absolute and relative amount. For instance, SCa elicited the accumulation of flavonol and flavone, tyrosol and phenolic acid equivalents in RS, whereas SK increased flavone and tyrosol equivalents. These variations also have functional relevance. These classes have been extensively described to have a wide range of health benefits in humans, such as preventive effects against aging and cardiovascular diseases, gut dysbiosis, diabetes, and cognitive impairment (Liu et al., 2017; Ma and Chen, 2020; Zhang et al., 2021). The specific increases in phenolics were more evident in RS, and in general, involved more frequently flavonoids than other phenolics. Nonetheless, some more generalized responses could be observed, since Mg elicited flavonol equivalents in both lettuce genotypes. Among dietary flavonoids, flavonols and their gut metabolites are described to have potential health benefits because of their antioxidant, cardioprotective, and anticancer activity (Barreca et al., 2021). Noteworthy, cyanidin equivalents were poorly represented and not affected by the treatments, despite the two genotypes differed for their pigmentation. Similarly, lignans, namely an emerging class of phenolics having estrogenic activity and being implicated in the modulation of the gut-brain axis (Senizza A. et al., 2020), were not affected by the treatments. As expected, the *in vitro* antioxidant capacity was consistent with the polyphenolic accumulation, in particular concerning TPC and metal reduction antioxidant assays (FRAP and CUPRAC). Nonetheless, anthocyanins, tyrosols, phenolic acids, and stilbenes were correlated with DPPH radical scavenging, and phosphomolybdenum activity values in the RS variety (Radovanović and Radovanović, 2010; Mathew et al., 2015; Vo et al., 2019). Tyrosol derivatives were the only phenolic class that was in correlation with antioxidant capacity in the GS (mainly with ABTS, CUPRAC, and metal chelating ability). Considering the inhibitory activities, flavones and lignans showed significant correlation coefficients for the alpha-amylase inhibitory activity of GS lettuce. Accordingly, flavonoids have been widely studied for their inhibitory potential toward amylolytic enzymes, and this functional role could represent a valid strategy to design functional foods and/or to manage type II diabetes (Giuberti et al., 2020). On the other hand, phenolics in the RS were loosely correlated to the enzymatic activities measured, except for lignans, whose amount was significantly correlated with AChE inhibitory activity. Overall, the genotype-related different modulation of the phytochemical profile was reflected in different correlations between phenolics and *in vitro* assays.

## Conclusion

This work illustrated how endogenous and exogenous factors, beyond stress (e.g., salinity, drought, heat, light intensity, etc.) (De Pascale et al., 2005; Mahouachi, 2007; Hazrati et al., 2017; Song et al., 2020), affect the efficiency by which plants accumulate minerals in leaves and produce dry biomass in relation to the available nutrients. Our data also demonstrated the range of variation that can be obtained by concurrently varying the concentration of macrocations in the NS, and the significant intraspecific variability in lettuce. The data provided evidence of the trade-off existing between total accumulation and mineral UtE, as well of the considerable interactions of macrocations when plants grow under non-limiting conditions. The alteration of the investigated macroelements' content significantly affected the morphology, physiology, quality, and functional properties of the edible product. The flexibility of the plant response and the large intraspecific variation to the different ratios of macrocations were adequate to provide specific phytochemical profiles. In particular, the RS cultivar conditions provided under controlled conditions more interesting responses considering both the compositional and functional properties. The observed wide-ranging effects on lettuce indicated that ensuring the perfect amount of minerals is not trivial when both plant and human nutritional needs are taken into account. A sustainable improvement (i.e., not based on increasing the use of fertilizers) of the mineral content of the lettuce could be obtained alongside a significant and cultivar-specific shaping of both phenolic compounds and health-related activities. Nonetheless, it is advisable that such a specific modulation of functional properties should be guided by the need of defined functional profiles and related consumer demand.

## Data Availability Statement

The raw data supporting the conclusions of this article will be made available by the authors, without undue reservation. The raw dataset on mass spectrometric characterization of phenolic compounds is provided as Supplementary Material.

## Author Contributions

GC and YR: conceptualization and visualization. BM-M, BS, GZ, and CE-N: methodology, validation, and formal analysis. BM-M and BS: software. GC, VDM, LL, GZ, SDP, and YR: investigation, writing—review, and editing. YR and SDP: resources. GC, VDM, LL, BM-M, BS, GZ, CE-N, SDP, and YR: data curation. GC, VDM, LL, and YR: writing—original draft preparation. GC, SDP, and YR: supervision. YR: project administration. All authors contributed to the article and approved the submitted version.

## Conflict of Interest

The authors declare that the research was conducted in the absence of any commercial or financial relationships that could be construed as a potential conflict of interest.

## Supplementary Material

Supplementary Figure 1 Light microscopy views of cross sections of leaf lamina of green **(A,C,E)** and red **(B,D,F)**. Salanova plants subjected to the three nutrient solutions (NSs): SCa **(A,B)**, SK **(C,D)**, and SMg **(E,F)**. Images are at the same magnification. Bar = 100 mm.

Supplementary Table 1 Ultra-high performance liquid chromatography coupled to a quadrupole-time-of-flight (UHPLC-QTOF) mass spectrometry analysis of polyphenols.

Supplementary Table 2 Correlation analysis among polyphenols and enzymatic activities.
