# Supplementary figures and images for: Isosmotic Macrocation Variation Modulates Mineral Efficiency, Morpho-Physiological Traits, and Functional Properties in Hydroponically Grown Lettuce Varieties (Lactuca sativa L.)
Source: Front Plant Sci. 2021 Jun 4;12:678799. doi: 10.3389/fpls.2021.678799 (PMC8212932; doi:10.3389/fpls.2021.678799)

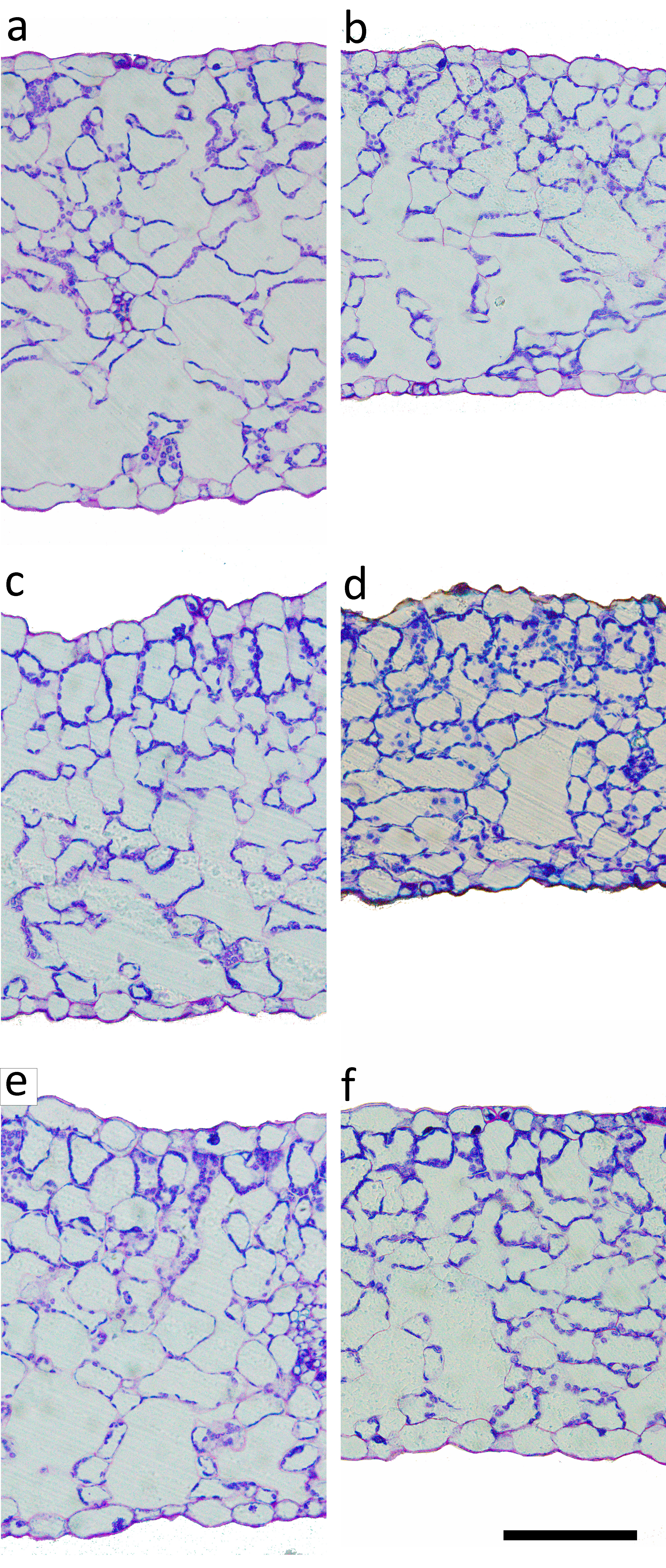

Supplement: Supplementary Figure 1 — Light microscopy views of cross sections of leaf lamina of green (A,C,E) and red (B,D,F). Salanova plants subjected to the three nutrient solutions (NSs): SCa (A,B), SK (C,D), and SMg (E,F). Images are at the same magnification. Bar = 100 mm. [file Image_1.JPEG]
